# Integrating imaging and genomics in prenatal Treacher Collins syndrome: evidence for practice and policy

**DOI:** 10.1186/s13023-025-04094-4

**Published:** 2026-03-19

**Authors:** Chunling Li, Wei Huang, Zhongzhi Gan, Yin Ling, Liyan Qiu, Yuanling Xiao, Fu Xiong, Fang Yang

**Affiliations:** 1 Department of Foetal Medicine and Prenatal Diagnosis, Zhujiang Hospital, Southern Medical University, 253 Industrial Avenue, Guangzhou, Guangdong 510282 China; 2 Department of Gynaecology and Obstetrics, Yunfu People’s Hospital, Yunfu, Guangdong China; 3 Department of Medical Genetics, School of Basic Medical Sciences, Southern Medical University, Guangzhou, Guangdong China; 4 Department of Ultrasound, Guangzhou Eighth People’s Hospital, Guangzhou Medical University, Guangzhou, Guangdong China

**Keywords:** Treacher Collins syndrome, TCOF1, Prenatal diagnosis, Genetic counselling, Ethical dilemmas, Mosaicism

## Abstract

**Background:**

To elucidate the prenatal diagnostic challenges, genetic landscape, and clinical outcomes of Treacher Collins syndrome (TCS), focusing on the role of *TCOF1* variants, prenatal ultrasound findings, and counselling implications.

**Methods:**

A systematic literature review (2000–2025) identified 273 TCS cases, supplemented by two index cases(one from each of two unrelated families) from our centre. Data included genetic testing (whole-exome sequencing, Sanger sequencing), prenatal imaging, and clinical outcomes. Variants in *TCOF1* were analyzed alongside genotype-phenotype correlations and mosaicism.

**Results:**

We assembled 275 patients in total, including 273 cases from the literature and two index cases from our centre. We summarized 15 individuals with prenatal ultrasound assessment and/or pregnancy outcomes(2 from our centre and 13 from the literature). Across 303 TCOF1 variant entries extracted from the included reports frameshift changes were most frequent at 66.01%, followed by nonsense variants at 10.56%, with missense, large deletions, and splice variants comprising the remainder. Pathogenic *TCOF1* variants were dominated by frameshift and nonsense changes, and truncations in later exons, for example exon 24, were enriched among severe phenotypes; overall, 68 of 275 cases, that is, 24.73%, were categorized as severe. Parental mosaicism was identified or suspected in 6 of the 275 cases (2.18%), including low-level paternal mosaicism confirmed in blood and semen in one family. Termination of pregnancy (ToP) occurred in 6 cases within this prenatal findings dataset, attributable to severe airway risks or parental choice. Postnatal management (airway support, hearing aids, reconstructive surgery) supported survival in 263/275 (95.64%) of all reported cases.

**Conclusions:**

Prenatal diagnosis of TCS relies on ultrasound detection of craniofacial anomalies and gene testing, but severity prediction remains challenging due to mosaicism and variable expressivity. Low-level parental mosaicism materially affects recurrence-risk counselling and should be actively considered when a foetal variant appears de novo. Early genetic diagnosis and personalized counselling are crucial for effective TCS management, helping families navigate medical, ethical, and sociocultural concerns.

**Supplementary Information:**

The online version contains supplementary material available at 10.1186/s13023-025-04094-4.

## Background

Treacher Collins syndrome (TCS, OMIM 154500), a craniofacial disorder, is among the most common forms of Mandibulofacial Dysostosis (MFD). The disease was named after Edward Treacher Collins, an English surgeon who extensively documented its characteristics in 1900. Currently, TCS prevalence stands at 1/50,000 live births, and there are four TCS subtypes, each with distinct clinical manifestations: TCS1 (OMIM# 154500), TCS2 (OMIM# 613717), TCS3 (OMIM# 248390), and TCS4 (OMIM# 618939). These subtypes result from variations in the *TCOF1*, *POLR1D*, *POLR1C*, and *POLR1B* genes. A recent systematic evaluation reported *TCOF1* as the primary gene in the Chinese TCS population [1], with 40% of TCS cases having a familial background, while the remaining 60% occur spontaneously [2].

Clinically, TCS is marked by bilateral craniofacial anomalies, including malar and mandibular hypoplasia (leading to malar flattening and micrognathia), downward-slanting palpebral fissures, lower eyelid colobomas, and external ear malformations (microtia or atresia of the external auditory canals). Approximately half of individuals affected experience conductive hearing loss, yet inner ear and cognitive development typically remain intact [3]. The severity ranges from mild, nearly undetectable traits to profound mandibular underdevelopment, which can cause life-threatening neonatal airway compromise [4]. Multidisciplinary management-encompassing airway support, feeding assistance, and craniofacial reconstruction-often enables individuals with TCS to achieve normal lifespan and intellectual function, underscoring its wide phenotypic variability but generally favourable prognosis.

Prenatal TCS diagnosis hinges on ultrasound imaging and genetic testing. Routine foetal ultrasound may reveal micrognathia, hypoplastic zygomatic arches, ear anomalies, cleft palate, and occasionally polyhydramnios, especially in severe cases [5]. Three-dimensional ultrasound can highlight subtle facial dysmorphologies, aiding differentiation from conditions like Pierre Robin sequence. However, mild TCS may escape detection, as illustrated by missed diagnoses in sporadic cases. When a familial *TCOF1* variant is known, targeted prenatal testing (via chorionic villus sampling or amniocentesis) can confirm TCS but cannot predict disease severity [6]. This variability complicates genetic counselling.

Ethical challenges emerge when TCS is identified prenatally. Although not typically lethal, severe manifestations involve multiple surgeries and prolonged care. Some parents may choose to terminate the pregnancy after assessing the potential severity of TCS, whereas others, informed by the generally favourable outcomes and lived experiences of TCS patients, may decide to continue, noting that a fulfilling life remains attainable. The decision is further influenced by social, cultural, and personal values, making prenatal TCS counselling especially delicate.

Moreover, several studies highlight the role of mosaicism in TCS, where low-level variants can produce minimal signs in one individual yet lead to severe disease in offspring, adding complexity to prenatal risk assessment [5, 7, 8]. To elucidate these decision-making processes, we combined data from our centre with a literature review of reported prenatal TCS cases, as detailed below.

## Methods and materials

### Literature search

A systematic literature search was conducted in PubMed and Scopus from January 2000 to March 2025, combining the following search terms using Boolean operators (“AND” or “OR”): “Treacher Collins syndrome,” “*TCOF1*,” “prenatal diagnosis,” “mandibulofacial dysostosis,” “micrognathia,” “microtia,” “hearing loss,” “frameshift,” and “nonsense mutation.” Only studies reporting human cases of Treacher Collins syndrome (TCS) with confirmed or presumed *TCOF1* variants were included. Conference abstracts, reviews, and studies lacking sufficient clinical or genetic details were excluded. The detailed selection process is illustrated in the PRISMA flow diagram (see Supplementary Fig. S1). The overall inclusion criteria were: (1) reported *TCOF1*-related variants (including but not limited to variants in exons or promoter regions) and (2) availability of comprehensive clinical information, with particular attention to prenatal diagnostic data. After applying the inclusion and exclusion criteria, 50 publications were ultimately included in our study.

### Study Participants and Family Cohorts

In addition to the cases identified through the literature search, we included a single-centre prospective study involving two Chinese families clinically diagnosed with TCS, conducted at Zhujiang Hospital of Southern Medical University. This study was approved by the Ethics Committee of Zhujiang Hospital of Southern Medical University, and informed consent was obtained from all participants for the publication of findings. Importantly, for the quantitative pooled analyses in this paper, a “case” was defined as one affected individual (fetus/neonate/child/adult) with a reportable TCOF1 variant and extractable clinical information. Therefore, only two index cases from our centre (one index case per family) were included in the pooled case-count analyses, together with 273 literature cases, yielding 275 cases in total (Supplementary Table 1). Additional relatives in the two families were evaluated to support segregation/mosaicism interpretation and to illustrate intrafamilial variability (e.g., pedigrees/figures), but were not counted as additional pooled cases. In Family 1, peripheral venous blood and available tissue samples were collected from the proband, parents, siblings, and other accessible relatives. Detailed clinical data from the proband, including findings from physical examinations, imaging studies such as temporal bone CT, and photographic documentation, as well as physical examination results from other relatives who declined additional tests like temporal bone CT, were gathered for comprehensive phenotypic characterization. In Family 2, a 27-year-old pregnant woman (gravida 2, para 1) underwent prenatal ultrasound that suggested TCS-related anomalies. Amniocentesis was performed at approximately 29–30 weeks of gestation, and foetal tissue samples were obtained following pregnancy termination. Peripheral blood samples were also collected from both parents. All samples were coded to ensure confidentiality, and detailed clinical, radiological, and genetic data from these families were systematically recorded in a dedicated database.

### Whole-exome sequence (WES), next-generation sequencing (NGS), and Sanger sequencing

In the first family, the proband, parents, and siblings, when accessible, had their peripheral venous blood and tissue samples collected. For the second family, amniocentesis was performed, and peripheral blood samples were collected from the parents for genetic testing. Genomic DNA was extracted with a validated kit (AmCare Genomics Lab, Guangzhou, China). Chromosomal structural abnormalities were identified through karyotyping. Genome copy number variations were detected via chromosome microarray analysis. We used WES to illuminate the monogenic disorder-associated single nucleotide changes. Genomic DNA was extracted from induced foetal tissue and peripheral blood with a validated kit (AmCare Genomics Lab, Guangzhou, China) and sequenced as paired-end 150-bp reads on the AmCareSeq-2000, targeting a mean on-target depth of 100–200× with >96% of targets covered at ≥20×. Raw reads were aligned to hg19 (GRCh37) using BWA v0.7.15, and processed with duplicate marking; variants were called by GATK HaplotypeCaller and annotated with VEP/ANNOVAR against gnomAD, ClinVar, and OMIM. Variants’ nomenclature was determined based on the *TCOF1* transcript NM_001371623.1, and classification followed ACMG/AMP guidelines. All candidate variants were confirmed by Sanger sequencing on an ABI 3730XL with primers designed in NCBI Primer-BLAST, and co-segregation was assessed in available relatives.

### Data extraction and statistical approach

Data were retrieved from all selected publications and extracted into a standardized data sheet as follows: (1) name of the first author; (2) year of publication; (3) country of origin; (4) patient age; (5) genetic information (including *TCOF1* variant type, cDNA position, exon location, and inheritance pattern); (6) prenatal findings; (7) outcome (e.g., pregnancy outcome, survival status); (8) any medical or surgical interventions and the corresponding results; (9) 20 TCS-related clinical features and, if reported, clinical severity; and (10) presence or absence of parental mosaicism. The clinical severity of TCS was assessed based on the scoring system developed by Teber et al. [9] and Vincent et al. [10], classifying a patient as mild if the total score was ≤ 8 and severe if the total score was ≥ 9, according to the criteria outlined by Ulhaq et al. [1]. Given the heterogeneity and incompleteness of case-based reports, we prespecified a descriptive analysis, no formal hypothesis testing was undertaken.

## Results

### Overall cohort description

We assembled a pooled cohort of 275 individuals with TCOF1-related Treacher Collins syndrome, including 273 literature casesreported in 50 publications from 19 different countries and two index cases from our center (Supplementary Table 1). This cohort contained 32 cases of foetuses or neonates, while the oldest adult patient was 50 years old. In total, 68 of the 275 cases (24.73%) presented with severe features (score ≥ 9). Mosaicism was confirmed or strongly suspected in 6 families; in each instance, the parent was either confirmed or presumed to carry a pathogenic *TCOF1* variant in a mosaic state.

### Prenatal findings and pregnancy outcomes

A total of 15 Treacher Collins syndrome (TCS) cases were included in the study on prenatal findings and pregnancy outcomes, which consisted of two index cases from our center (one from each of two unrelated families) and 13 additional cases drawn from the literature. Reported pregnancy termination occurred in 6/15 (40.00%) cases as summarized in Table 1. Prenatal ultrasound findings were explicitly described for 11 of 15 individuals; for the remaining cases, pregnancy-outcome information was available but detailed prenatal ultrasound descriptions were not reported. All cases harbored pathogenic variants in the *TCOF1* gene, most of which were frameshift or nonsense changes, though a few large deletions (Cases 9, 11, and 12) or a splice-site variant (Case 7) were also observed. Consistent with classical TCS, most patients, whether diagnosed prenatally or postnatally, exhibited malar hypoplasia, micrognathia or mandibular underdevelopment, external auditory canal atresia, microtia, and downward-slanting palpebral fissures.

**Table 1:** Individuals included in the analysis of prenatal ultrasound findings and/or pregnancy outcomes in TCOF1-related TCS (n=15)

**No**	**Study(author)**	**Year**	**Country**	**Case No.**	**Sex**	**Age at last examination(yr, otherwise indicated)**	**Prenatal findings**	**Molecular anomaly**	**cDNA position**	**Exon**	**score**	**Survival/Pregnancy outcome**	**Treatment/Management**	**Reference**
1	Our study	2025	China	1	F	Neonate	None	frameshift, premature stop	chr5:149771658-149771661 delAGTG	20	7	No (postnatal death from severe respiratory distress)	None	
				2	F	Fetus(33+1 weeks)	Polyhydramnios, micrognathia, microtia, bilateral echogenic auricles, cleft palate	p.S1021Afs*6	c.3054_3060dup	20	6	No (pregnancy terminated)	None	
2	Fan et al	2024	China	3	ND	12	Not reported	p.K1457Efs*12	c.4369_4373delAAGAA	24	9	Yes (alive)	Multiple craniofacial surgeries and bone bridge implantation, favorable outcome	[11]
				4	ND	Fetus(33+1 weeks)	Unilateral microtia and facial anomalies	p.K1457Efs*12	c.4369_4373delAAGAA	24	ND	No (pregnancy terminated)	None	
				5	M	Adult	Not reported	p.K1457Efs*12	c.4369_4373delAAGAA(suspected)	24	1	Yes (alive)	None	
3	Tandon et al	2024	USA	6	M	Neonate(38 6/7 weeks)	Micrognathia, low-set ears	p.Arg897*	c.2689A>T	17	9	Yes (alive)	NG-tube feeding, stable on room air	[12]
4	Wang et al	2023	China	7	ND	Fetus(25 weeks)	Polyhydramnios, absent nasal bone, suspected glossoptosis	Splice-site mutation causing aberrant mRNA splicing	c.1894-2A > T	?	8	No (pregnancy terminated)	None	[5]
5	Wang et al	2023	USA	8	ND	Fetus(38 weeks (Cesarean delivery))	No anomalies on ultrasound at 20 and 30 weeks	p.Leu1225Alafx*16	c.3671dup	23	8	Yes (alive)	Tracheostomy, PEG placement, multidisciplinary care	[13]
6	Chou et al	2022	China	9	ND	Fetus(28 weeks)	Receding chin, micrognathia, downward-slanting palpebral fissures	Microdeletion at arr 5q32(147,204,320–149,778,916)x1			4	No (pregnancy terminated)	None	[14]
7	Zeng et al	2021	China	10	M	Neonate(39+4 weeks)	Polyhydramnios	p.W541*	c.1622G>A	11	10	Yes (alive)	None	[15]
8	Liu et al	2020	China	11	M	Fetus(24–25 weeks)	Asymmetric nasal bone, small auricles	p.(?)	ND	Deletion of exons 2-6	7	No (pregnancy terminated)	None	[16]
				12	M	39	Not reported	p.(?)	ND	Deletion of exons 2-7	6	Yes (alive)	None	
9	Konstantinidou et al	2013	Greece	13	M	Fetus(25 weeks)	Hypoplastic mandible and nasal bones, cleft palate, abnormal earlobes		c.1729C>T	11	8	No (pregnancy terminated)	None	[17]
10	Bauer et al	2013	USA	14	F	Neonate(36 weeks)	Not reported	p.1456Thrfs*18	c.4355_4356ins14	24	9	Yes (alive)	Multiple craniofacial surgeries (e.g., cranial vault remodeling, oromandibular repair), bone conduction hearing, sign language	[18]
11	Su et al	2007	Taiwan	15	F	Neonate	Polyhydramnios, growth restriction	p.741 Q>	c.2221C> T	14	11	No (postnatal death)	None	[19]

Note: Table 1 includes fetuses/neonates with prenatal ultrasound assessment as well as affected relatives reported in the same pedigrees to contextualize recurrence and variable expressivity; prenatal ultrasound details were not available for some relatives (marked as “Not reported”). Abbreviations: ND, Not determined / not described; ToP, termination of pregnancy; NG, nasogastric; PEG, percutaneous endoscopic gastrostomy; IFA, inferior facial angle; NA, not applicable; ND, not determined

**Fig. 1:**
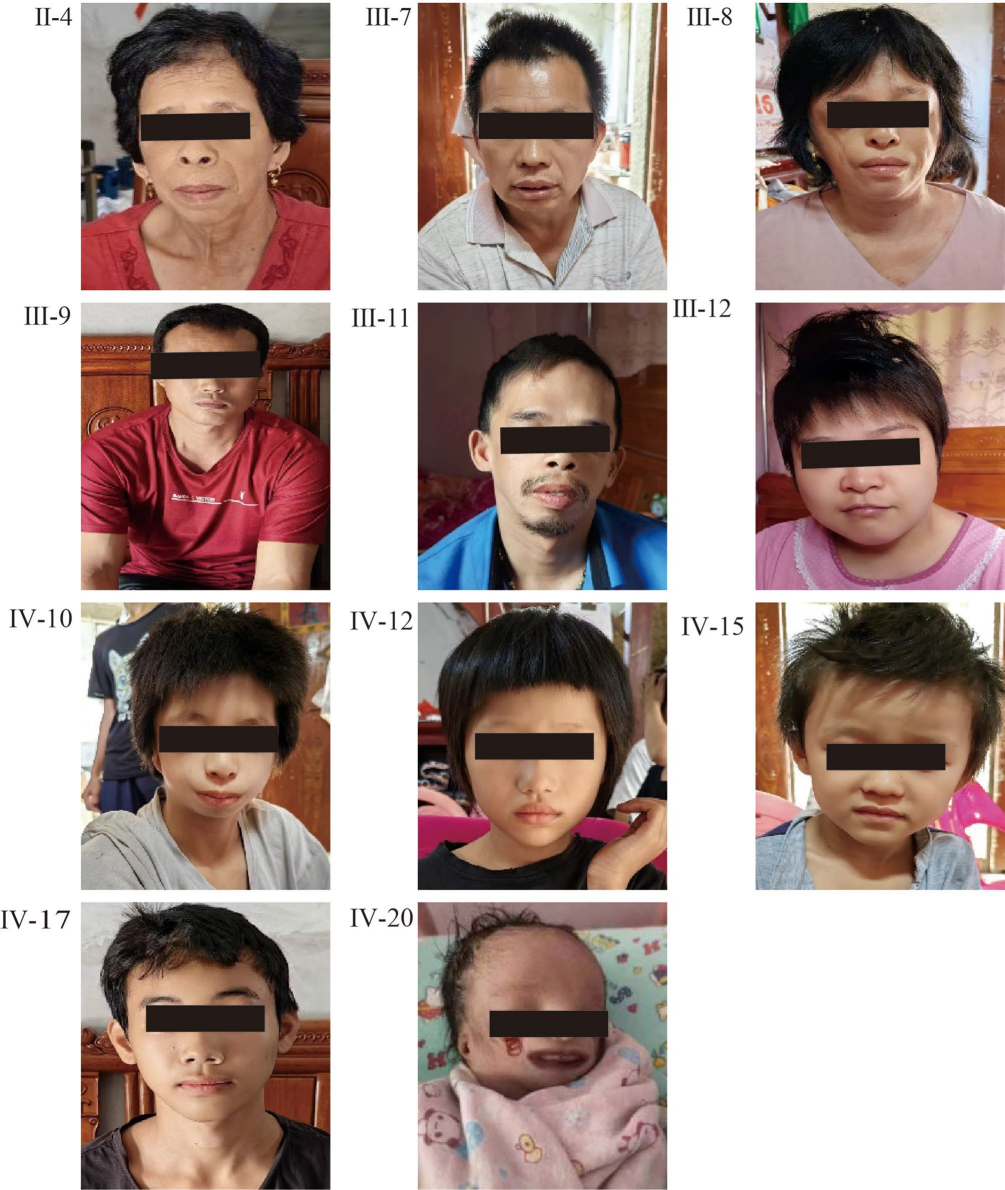
Facial features of some members of the first family. Common characteristics of the affected individuals include malar and mandibular hypoplasia, downward-slanting palpebral fissures, microtia, and lower eyelid coloboma. These features were observed in the neonatal period for one individual (IV-20)

Family 1 spans four generations with 39 individuals, among whom 13 (nine males, four females) were clinically diagnosed with TCS. These relatives were evaluated for segregation and phenotypic description only and were not counted as additional cases in the pooled 275-case analyses. Facial deformities were variably expressed, ranging from severe mandibular hypoplasia and external ear canal atresia to milder ear anomalies. Notably, the proband (IV-20) presented with a lower eyelid coloboma and died shortly after birth due to profound respiratory compromise (see Supplementary Table S2). Molecular testing in 11 family members (plus one tissue sample) uncovered a novel heterozygous frameshift (chr5:149771658–149771661 delAGTG) that was absent from known genomic databases. Five patients (including the proband) carried this variant, Sanger sequencing did not detect the variant in the parental peripheral blood samples of the proband, therefore low-level parental mosaicism cannot be excluded, and the variant showed familial segregation in other affected relatives (see Supplementary Fig. S2). Unfortunately, the remaining family members declined further examinations, such as temporal bone CT, which limits full clinical characterization (Fig. 1; Supplementary Fig. S3).

**Fig. 2:**
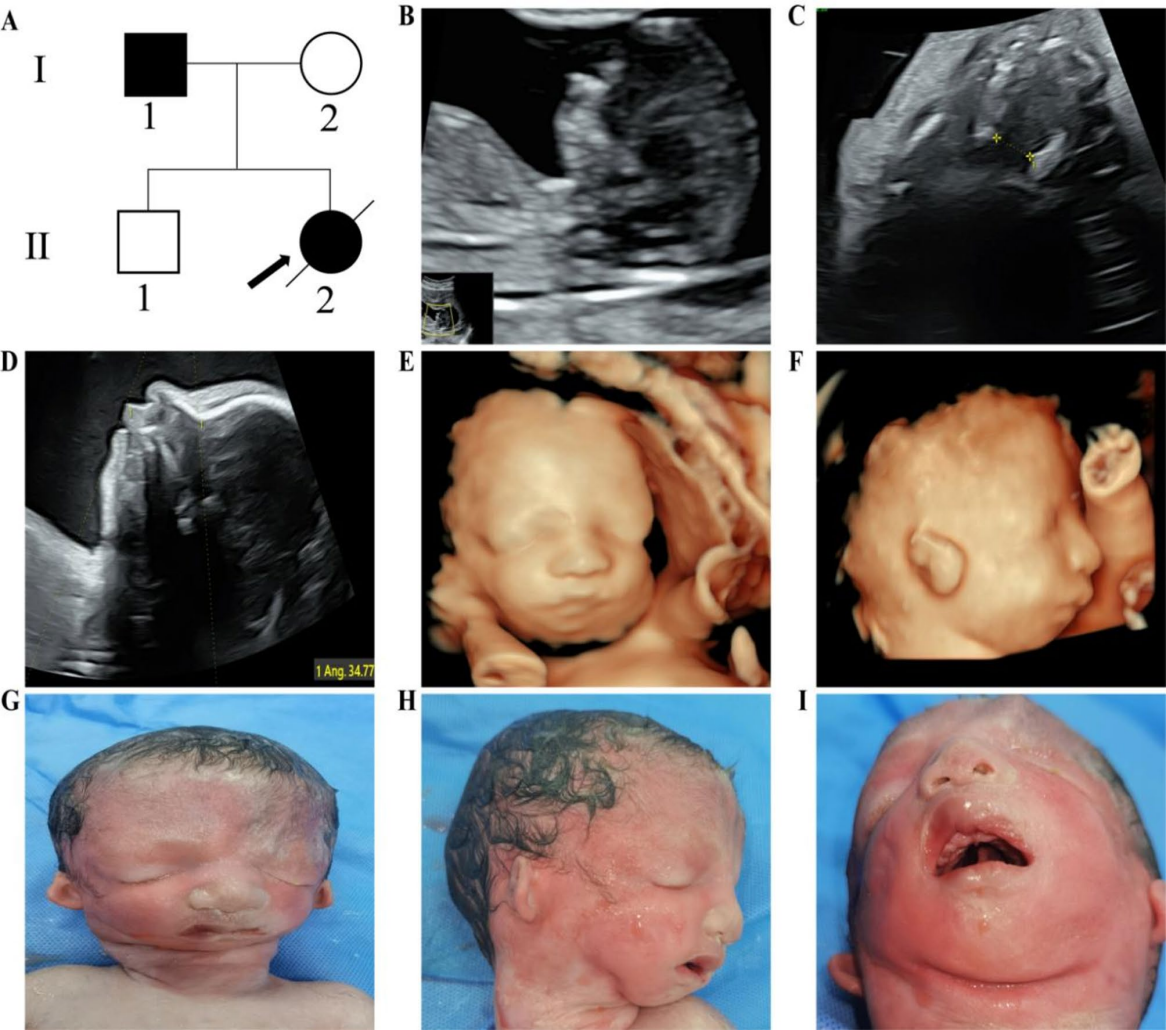
A female foetus was assessed for craniofacial features using 2D and 3D ultrasonography at 29 weeks of gestation considering the family degree; (**A**) Family pedigree, with II-2 as the proband, and I-1 showing somatic mosaicism, gonadal mosaicism; (**B**) NT examination revealed micrognathia, with the 2D and the rendering mode of 3D ultrasonography at 29 weeks of gestation (both left and right) showing a cleft palate (**C**), micrognathia (**D**), and symmetrical down-slanting palpebral fissures and microtia (**E**, **F**). The craniofacial malformation of the aborted foetus at 33 weeks, exhibiting symmetrical downward-slanting palpebral fissures, abnormal ears, micrognathia, and a cleft palate (**G**, **H**, and **I**)

Family 2 comprised a 27-year-old pregnant woman (gravida 2, para 1) with an initially unremarkable first-trimester screening. However, at 28 weeks of gestation, detailed two- and three-dimensional ultrasound revealed polyhydramnios, micrognathia, microtia, bilateral abnormal high-echogenic auricles, and a cleft palate, strongly suggesting TCS. Additional molecular analyses (karyotyping, CMA, and WES) confirmed a *de novo* frameshift variant in exon 20 of *TCOF1*-c.3054_3060dup (p.S1021Afs*6)-in the foetus. Subsequent targeted testing demonstrated paternal low-level mosaicism (~ 10.84% in blood, ~ 8.74% in semen). After extensive genetic counselling, the parents elected to terminate the pregnancy at 33 + 1 weeks. Post-delivery examination confirmed marked craniofacial features consistent with TCS features, mirroring the prenatal ultrasound images (Fig. 2).

Thirteen additional cases were collected from published reports, many exhibiting a similar spectrum of frameshift or nonsense *TCOF1* variants with variable prenatal ultrasound findings. Of note, two further examples of paternal mosaicism were identified, corroborating our observation in Family 2 that a subtly or asymptomatic father can harbor low-level *TCOF1* variants and transmit severe TCS to offspring. In line with prior studies, later-exon frameshift or nonsense variants (e.g., in exons 20 or 24) frequently correlate with higher scores (≥ 7) and more obvious prenatal ultrasound findings (Case 14, Case 15). Nonetheless, identical variants can manifest variable expressivity: for example, c.4369_4373delAAGAA in the same family may yield mild phenotypes in one individual but severe anomalies in another, implying involvement of mosaicism, modifier genes, or other factors.

Nearly all foetuses with pronounced TCS-related craniofacial anomalies-such as overt micrognathia, cleft palate, ear deformities, and polyhydramnios-were either ToP (e.g., Case 2 in this study, Cases 7 and 9 reported by Wang and Chou [5, 14], respectively, and Case 13 by Konstantinidou [17]), or at significant risk of neonatal respiratory compromise if allowed to progress to term. Conversely, in instances with milder ultrasound anomalies or when parents were prepared for potential airway, feeding, and reconstructive interventions, some pregnancies were continued (e.g., Cases 6, 8, 10, and 14). Case 1 (Family 1) highlights the dangers of missed prenatal detection: a foetus with undiagnosed marked mandibular hypoplasia ultimately succumbed to critical respiratory compromise shortly after birth, emphasizing the importance of thorough foetal jaw assessment and early preparedness for neonatal airway management.

### Postnatal phenotypes, genotype-phenotype correlations, and clinical management

We identified a total of 275 unique TCS patients, including two index cases from our center and 273 cases from 50 published reports. Among these patients, 263 (95.64%) cases survived, whereas 12 (4.36%) either died shortly after birth due to critical airway compromise or underwent ToP. Notably, the compiled dataset included a total of 303 distinct TCOF1 variants, as multiple variants were occasionally observed in a single patient or within the same family. Among these 303 variants, the majority were frameshift variants (*n* = 200, 66.01%) or nonsense variants (*n* = 32, 10.56%). Additionally, 31 variants (10.23%) had missense variants, 8 variants (2.64%) represented large deletions, 7 variants (2.31%) were splice-site changes, and the remainder consisted of other or complex alterations, including rare in-frame deletions, potential promoter or UTR changes.

As expected, most truncating variants fell in the latter half of the coding region (exons ~ 11–24). However, we also observed examples of early-exon frameshifts (exons ~ 1–10) and large 5′ deletions leading to severe TCS phenotypes, underscoring that the position of the variant alone does not always predict clinical severity.

**Fig. 3:**
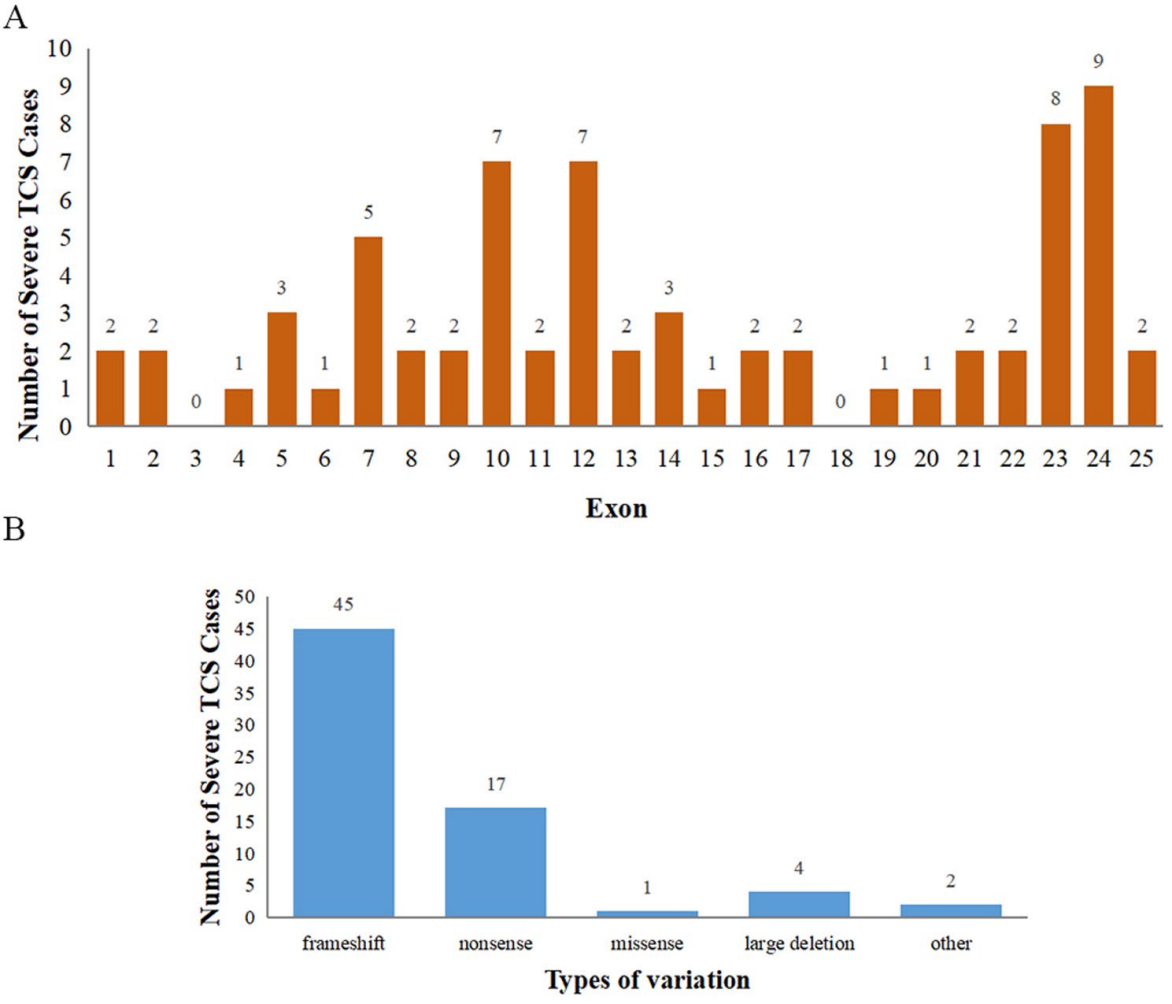
Distribution of severe phenotypes across different variant types and exon locations

Within this subgroup, 68 cases (24.73%) were scored as severe (≥ 9 points). Notably, several patients with frameshift variants in exons 23 and 24, such as exon 24 frameshift mutations, tended to show severe craniofacial abnormalities and airway compromise, which were reflected in higher clinical scores. Additionally, large deletions involving exons 2–7 were also frequently observed among patients with severe phenotypes, often resulting in profound craniofacial malformations and respiratory distress (Fig. 3). Furthermore, a subset of patients with splice-site variants or other complex alterations involving the 5’region, including large deletions of the 5’UTR, demonstrated variable severity, suggesting that even less common mutations can contribute to the overall clinical spectrum of TCS. Mosaic variants were documented in 6 cases (2.18% of total cases), with paternal mosaicism being the most common scenario. In such families, the apparently unaffected or minimally affected father carried a low-level frameshift mutation and produced offspring with moderate-to-severe TCS, illustrating the complex genetic counselling challenges in TCS.

Regarding clinical management, 47 (17.09%) patients have been reported to have received treatment. Conductive deafness was documented in 200 cases (72.73%); among the 37 patients with available treatment records, 22 showed clear improvement while the outcomes for the remaining cases were not explicitly described. These patients underwent hearing rehabilitation using bone-anchored hearing aids, which typically resulted in improved speech recognition. Cleft palate was present in 29 patients, of whom 6 were clearly reported to have undergone surgical repair in infancy or early childhood to facilitate oral feeding and optimize speech development. Mandibular distraction osteogenesis or other major craniofacial reconstructive procedures were performed in at least 6 severe cases, often in multiple stages. In addition, 39 cases exhibited intellectual or developmental disabilities, with scores generally ranging between 6 and 9; among these, 21 were reported to have received treatment, and the majority of treated patients demonstrated Favourable outcomes. Many mild and moderate cases avoided airway interventions altogether and benefited from audiological assessments, speech therapy, or minor external ear reconstruction. Among the 68 severe cases, only 3 deaths were reported, including 1 case in which treatment was ineffective, and a total of 14 cases were reported to have received treatment, with only 2 still requiring ongoing management, including 1 case of motor developmental delay. These findings indicate that despite the inherent challenges in genotype–phenotype correlations, most cases survived beyond the neonatal period, and a substantial proportion achieved functional improvement with multidisciplinary interventions encompassing airway management, feeding support, surgical reconstruction, and hearing rehabilitation.

## Discussion

TCS causes profound facial anomalies due to atypical development of the first and second branchial arches during weeks five to eight of gestation [20]. Our aggregated cohort of 275 cases, which included two index cases from together with two newly characterized Chinese families, allows a balanced appraisal of prenatal detection, mutational architecture, mosaicism, and practical counselling, while situating our observations alongside prior clinical and molecular series. Several themes emerge. First, truncating variants in *TCOF1* remain the dominant molecular finding across populations, but variant position alone is not a reliable predictor of clinical severity. Second, low‑level parental mosaicism is more consequential than its sparse reporting might suggest and directly influences recurrence‑risk counselling. Third, prenatal imaging identifies a recognizable pattern but may miss elements that become apparent postnatally, which underscores the value of integrating imaging with targeted molecular diagnosis and anticipatory care planning. With respect to the mutational spectrum, our findings accord with early and contemporary molecular studies that established loss‑of‑function changes in *TCOF1* as the primary cause of TCS, with *POLR1D*, *POLR1C*, and *POLR1B* accounting for a minority of cases [2, 10]. In our dataset, frameshift and nonsense variants were the most frequent classes, similar to patterns reported by Vincent et al. in a large multicentre series and by Teber et al. in an earlier genotype‑driven cohort [9, 10]. The distribution of truncating variants across exons 11 through 24 was common in our cases, and several severe cases carried frameshift changes in exons 23 or 24. This aligns with observations from other Chinese cohorts that have highlighted recurrent variants in late exons, although conclusions about prognostic value should be cautious because severe phenotypes were also seen with early‑exon frameshifts and with large proximal deletions [6, 7, 15, 20, 21]. In other words, our data support the view that later‑exon truncations appear enriched among severe presentations, yet there is wide overlap across the transcript, which limits the predictive utility of exon position for individual counselling.

The prenatal phenotype in our compilation was dominated by micrognathia and external ear anomalies, often accompanied by cleft palate or polyhydramnios, findings that are familiar from case reports and small series focused on foetal diagnosis [5, 14, 17, 21]. Micrognathia is a stable signal in mid‑gestation and late‑gestation imaging, but its quantitative assessment, for example with the inferior facial angle, is not uniform across studies and clinical centres [22]. Three‑dimensional ultrasonography can improve recognition of mandibular hypoplasia and auricular malformations and can help differentiate TCS from phenotypically overlapping conditions such as Pierre Robin sequence, as illustrated by foetal imaging reports and visual diagnosis case studies [23]. At the same time, some structures that are highly informative postnatally, such as external auditory canal atresia, are seldom captured in utero even with high‑quality imaging. Our dataset mirrors this pattern and strengthens the practical message that ultrasound and targeted genetic testing are complementary in suspected TCS.

In examining genotype to phenotype correlations, and consistent with earlier surveys, most *TCOF1* variants in our series were truncating changes, including small deletions or duplications that generate premature stop codons, which underscores the central role of haploinsufficiency in disrupting ribosome biogenesis, neural crest cell migration, and branchial arch development, as reported by Su et al. and others [2, 15, 19]. Frameshift changes in exons 23 and 24 appeared enriched among severe cases with marked airway compromise; however, early exon truncations and large 5′ deletions were also represented among severe presentations. Together, these observations caution against treating exon location as a deterministic predictor of severity, aligning with the view of Vincent et al. that genotype to phenotype relationships in Treacher Collins syndrome remain inconsistent [10]. Our data add weight to the message that counselling should present a spectrum of plausible outcomes rather than a deterministic forecast anchored to variant location.

The identification and quantification of low-level parental mosaicism in our cohort represents one of the most significant contribution. Symptoms of genetic mosaic diseases frequently present in the skin and brain, showcasing facial dysmorphism, asymmetric growth disruptions, and vascular abnormalities. Limited research has been conducted on the presence of mosaicism in sperm, and there are only a few studies that compare mosaicism levels between germline and somatic cells [24, 25], particularly there were only early descriptions, and until recently detection had been limited to clinical inference or low-resolution sequencing. In our overall series, we confirmed or strongly suspected parental mosaicism in six families. Notably, in Family 2, the clinically unaffected father carried low-level *TCOF1* mosaicism (~ 10.84% in blood and ~ 8.74% in semen), which led to severe TCS in his foetus. To our knowledge in TCS, this is the first documented case of low-level germline mosaicism identified via next-generation sequencing (NGS), whereas earlier studies (for example, Fan et al. [11]) could only infer mosaicism from family pedigrees. Previous work has described maternal or paternal mosaicism with varying levels of mutant alleles, but the true frequency of mosaicism is likely underestimated because very low-level variants may go undetected by standard Sanger sequencing. In our cohort, each low-level mosaic case correlated with moderate to severe TCS (one unscored individual with partial features, two individuals scoring 6, and three scoring 8 or higher). Such findings highlight the necessity of screening for parental mosaicism when both parents seem unaffected but a familial TCS variant is present. Since up to 80% of de novo mutations arise on the paternal haplotype [26], initial testing in fathers is practical, given the invasive nature of obtaining oocytes. Furthermore, identifying parental mosaicism has direct reproductive implications, as couples may opt for preimplantation genetic testing for monogenic conditions (PGT‑M) to avoid having a child with severe TCS.

In many countries, prenatal testing policies are increasingly judged against structured ethical frameworks: reproductive autonomy, proportionality, justice, and societal implications. For example, Kater-Kuipers et al. [27] showed that Dutch professionals explicitly link NIPT practice to these four pillars, stressing that informed choice through robust counselling is indispensable. Applying this to TCS, any offer of prenatal sequencing—particularly when a familial *TCOF1* variant is known or when ultrasound raises suspicion—should be embedded in pre-test discussions that cover prediction limits due to variable expressivity and parental mosaicism. Gullo et al. [28] further highlight that cell-free DNA–based innovations raise medicolegal concerns if results are misinterpreted as diagnostic, reinforcing the importance of confirmatory invasive testing before decisions are made. By situating TCS within this broader evidence-based policy discourse, our findings support standardized counselling checklists, equity of access irrespective of socioeconomic background, and clear separation of autonomy-aimed screening from preventive antenatal care. Clarity of institutional guidance is also crucial from a legal standpoint. Zaami et al. [29] emphasize that poorly defined protocols increase the risk of malpractice claims, ranging from “wrongful birth” to disputes over counselling omissions. The JAMA commentary on ambiguous guidelines [30] similarly warns that vague recommendations make it difficult to establish whether professional standards were met. For TCS, potential points of contention include failure to discuss the implications of paternal mosaicism, omission of genetic testing when hallmark ultrasound features such as micrognathia with auricular anomalies are present, or lack of planning for neonatal airway compromise. Zhytnik et al. [31] add that advanced paternal age increases the burden of de novo mutations, reinforcing the need for systematic policies on when to recommend extended screening. To reduce variability that courts may construe as negligence, national guidance should specify indications for sequencing, minimum consent elements, validation standards, and referral thresholds, while international statements should harmonize scope and equity principles.

Building on these observations, we offer several practice-oriented contributions. We expand the *TCOF1* variant spectrum with a novel frameshift identified in a multigenerational family and collate prenatal imaging features that are most consistently observed. We demonstrate that low‑level paternal mosaicism can be confirmed across blood and semen by high‑depth sequencing, which strengthens recurrence‑risk counselling and suggests a concrete testing sequence in suspected de novo cases. We frame exon position findings as an enrichment pattern with considerable overlap rather than as a deterministic rule, which we believe better reflects clinical reality. Finally, we translate these observations into clinical counselling messages that are directly actionable. For suspected TCS on ultrasound, referral for targeted molecular testing including trio analysis is reasonable. When a pathogenic *TCOF1* variant is identified in the foetus without parental features, deliberate search for parental mosaicism with adequate analytical depth should be considered, beginning with the father when feasible. Families should be advised that severity is difficult to predict and that delivery at centres with neonatal airway expertise can mitigate early risk. Early audiologic assessment and timely cleft and craniofacial interventions are likely to improve functional outcomes, which is consistent with the experience reported across multiple cohorts.

Several limitations qualify these findings. Our synthesis relied heavily on case reports, which may over-represent severe phenotypes and under-report milder cases. Mosaicism was only assessed in a minority of families, and very low-fraction variants may still evade detection even with deep sequencing. Our ethical and policy discussion draws on international frameworks that may not be universally applicable. Future studies should therefore build multicentre prospective cohorts with standardized severity scoring, harmonized mosaicism workflows, and systematic documentation of counselling outcomes. Integration of paternal semen testing into reproductive genetics could further clarify recurrence risks. Finally, longitudinal registries linking prenatal findings, genetic results, perinatal management, and long-term functional outcomes will be critical for evidence-based guideline development.

## Conclusion

In summary, our data reinforce a pragmatic approach to Treacher Collins syndrome in the prenatal setting. Ultrasound features such as micrognathia and auricular anomalies should prompt targeted molecular testing, yet variant position in *TCOF1* alone cannot predict severity. Low-level parental mosaicism, particularly in fathers, is clinically meaningful and should be actively assessed when a foetal variant appears *de novo*, using high-depth assays and, where feasible, semen testing. These findings translate into clear counselling messages: communicate uncertainty explicitly, plan delivery in airway-capable centres when risk is anticipated, and initiate early audiologic and craniofacial care after birth. At the programmatic level, services should standardize pre-test consent that explains analytic limits and confirmatory pathways, define referral triggers from foetal imaging to genetics, and document value-sensitive choices to ensure equity and medicolegal clarity. Future work should establish prospective, multicentre registries with harmonized severity scoring and mosaicism workflows, link prenatal data to long-term functional outcomes, and evaluate cost and feasibility of paternal mosaicism testing. By integrating careful imaging, disciplined genomics, and ethically robust counselling, prenatal care for Treacher Collins syndrome can be both scientifically rigorous and responsive to the realities facing families.

## Supplementary Information

Below is the link to the electronic supplementary material.

Supplementary Material 1

Supplementary Material 2

## Data Availability

This article does not publicly disclose its raw data due to concerns about protecting participant anonymity and adhering to policy. However, the datasets utilized and/or analyzed during this study can be provided by the corresponding author upon a reasonable request.
