# Supplementary material for: Integrating imaging and genomics in prenatal Treacher Collins syndrome: evidence for practice and policy
Source: Orphanet J Rare Dis. 2026 Mar 19;21:99. doi: 10.1186/s13023-025-04094-4 (PMC13001374; doi:10.1186/s13023-025-04094-4)
Supplement: Supplementary file 1 — Supplementary Material 1 [file 13023_2025_4094_MOESM1_ESM.docx]

**Supplementary materials**

**Supplementary Table S1** Characteristics of the 275 included TCS patients and/or references.

**Supplementary Table S2** Clinical features and genetic findings for the two new TCS families description


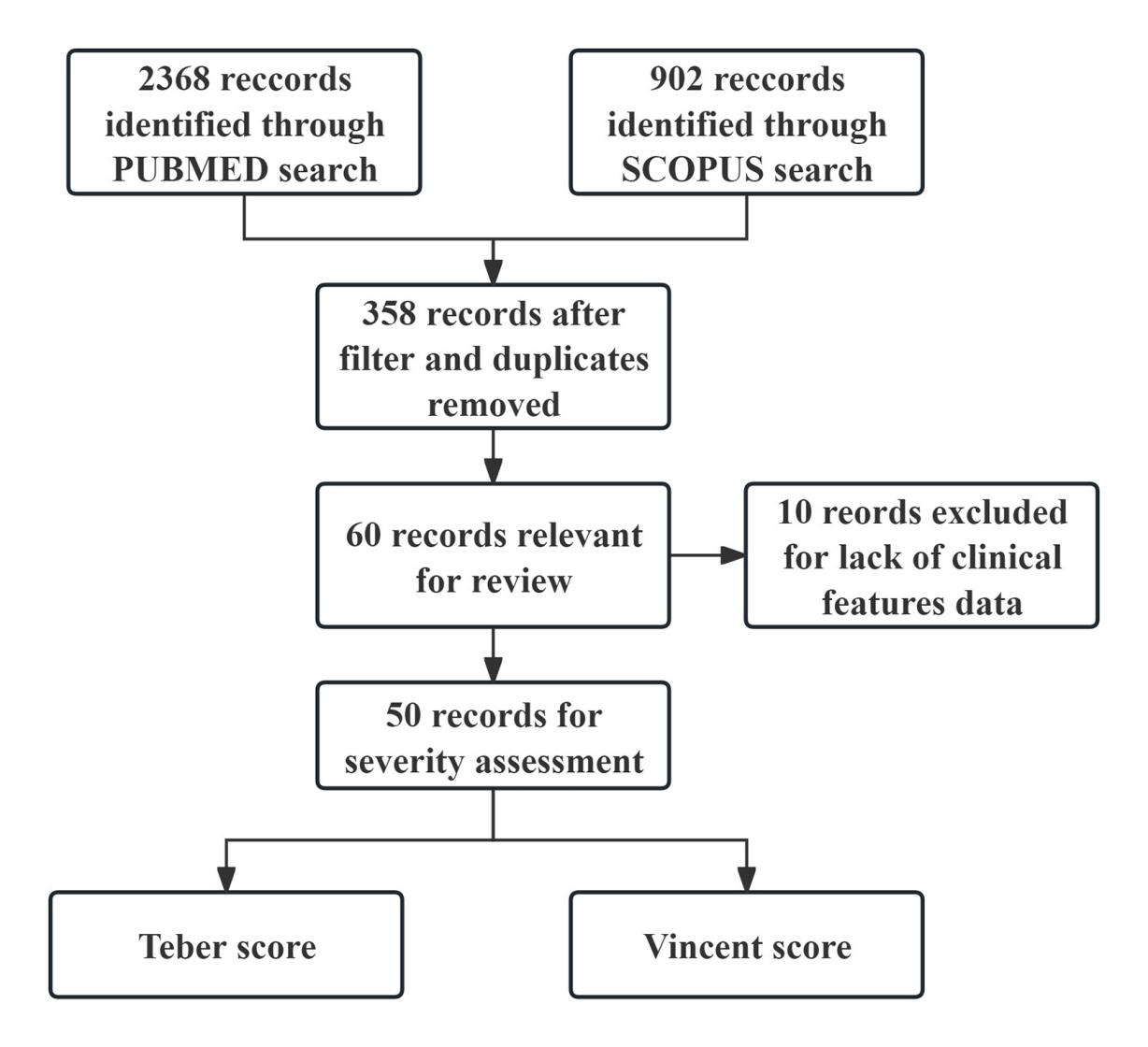


**Supplementary Fig. S1** Flow diagram (PRISMA) illustrating the literature search and selection process.


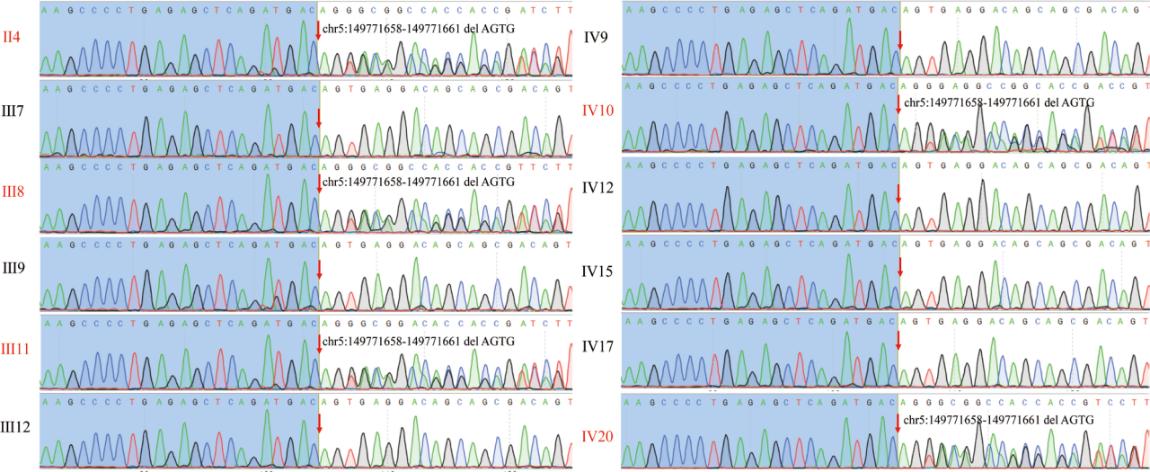


**Supplementary Fig. S2** Sequencing electropherograms generated using genomic DNA from circulating leukocytes. II-4, III-8, III-11, IV-10, IV-20 were affected with TCS and harbored the heterozygous deletion chr5:149771658-149771661 delAGTG in the *TCOF1* gene.


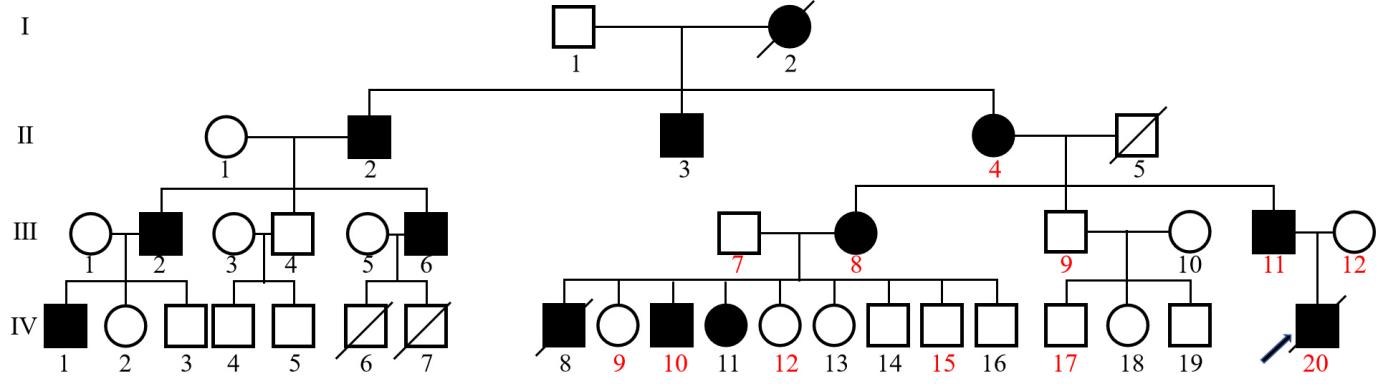


**Supplementary Fig. S3** Family pedigree, including members with inherited TCS. Squares, circles, and diagonal lines represent male, female, and deceased members, respectively. The arrow indicates the proband foetus. Open and filled colour symbols indicate unaffected and affected members, respectively. Among the 39 individuals, 13 were affected to varying degrees. Note: I-2, II-5, IV-6, IV-7, and IV-20 are deceased.
